# A review of compensatory growth following lysine restriction in grow-finish pigs^1^

**DOI:** 10.1093/tas/txaa014

**Published:** 2020-02-03

**Authors:** Mariana B Menegat, Steve S Dritz, Mike D Tokach, Jason C Woodworth, Joel M DeRouchey, Robert D Goodband

**Affiliations:** 1 Department of Diagnostic Medicine/Pathobiology, College of Veterinary Medicine, Kansas State University, Manhattan, KS; 2 Department of Animal Sciences and Industry, College of Agriculture, Kansas State University, Manhattan, KS

**Keywords:** amino acid restriction, catch-up growth, compensatory growth, swine

## Abstract

Compensatory growth induced by lysine (Lys) restriction in grow-finish pigs is a complex physiological process affected by many factors and interactions, principally genotype, stage of growth at restriction, nature of nutritional restriction, and patterns of restriction and recovery. The scarcity of standard comparisons across the literature has hindered the characterization of important determinants of compensatory growth. Therefore, the present publication aims to review the current state of knowledge on compensatory growth induced by Lys restriction in grow-finish pigs, develop a database from peer-reviewed literature to standardize comparisons to characterize the occurrence of compensatory growth, and provide practical considerations for compensatory growth under field conditions. The literature search focused on publications directly or indirectly evaluating compensatory growth by having a period of Lys restriction followed by a recovery period of Lys sufficiency for grow-finish pigs. The database included 14 publications and 57 comparisons expressed as relative differences of restricted pigs compared to nonrestricted pigs. The database analysis described compensatory growth into complete, incomplete, and no compensatory growth categories and characterized the patterns of restriction and recovery in each category. The review of literature and database analysis supports the occurrence of compensatory growth induced by Lys restriction in grow-finish pigs. The degree of Lys restriction and duration of restriction and recovery periods seem to be critical in explaining differences between complete and incomplete compensatory growth, whereas Lys level in the recovery period seems to be critical between incomplete or no compensatory growth. Compensatory growth seems to be more likely if: 1) the degree of Lys restriction is between 10% and 30%; 2) Lys restriction is induced before pigs reach their maximum protein deposition; 3) duration of Lys restriction is short (maximum 40–45% overall duration) and duration of recovery period is long (minimum 55–60% overall duration); and 4) Lys level in recovery is close to or above the estimated requirements. In addition, compensatory growth can occur under commercial conditions and there seems to be an opportunity to exploit compensatory growth in grow-finish pigs to reduce feed cost and improve feed efficiency under certain market conditions.

## INTRODUCTION

Compensatory growth is defined as a physiological process whereby animals undergo a period of accelerated growth rate following a period of restricted growth (Hornick et al., 2000). Growth restriction is typically induced by nutritional depletion and seems to be the primary requisite for compensatory growth to occur (O’Connell et al., 2006). Lysine (Lys) depletion is commonly known to have a considerable impact on the growth performance of lean pigs because Lys is the first limiting amino acid in most swine diets (NRC, 2012). Compensatory growth induced by Lys restriction in grow-finish pigs has been described in the literature (Chiba et al., 2002; Fabian et al., 2004; Reynolds and O’Doherty, 2006; Suárez-Belloch et al., 2015), but the response is not consistent (Chiba et al., 1999; Fabian et al., 2002; Cloutier et al., 2016). Compensatory growth is a complex phenomenon affected by a number of factors and interactions, for instance, genotype, stage of growth at restriction, nature of nutritional restriction, and patterns of restriction and recovery (Wilson and Osbourn, 1960). To date, the variation in methodology and scarcity of standard comparisons across the compensatory growth literature have hindered the characterization of important determinants of compensatory growth in grow-finish pigs.

The interest of the swine industry in compensatory growth predominantly lies in the potential to improve swine production efficiency. Strategies to exploit compensatory growth induced by Lys restriction aim at the improvement of Lys and nitrogen utilization for lean growth and, consequently, reduction of nitrogen excretion in the environment (Whang et al., 2003; Fabian et al., 2004; O’Connell et al., 2006). Moreover, the high cost of protein sources favors the exploitation of compensatory growth induced by Lys restriction to allow reductions in feed cost and improvements in feed efficiency.

Thus, the present publication aims to review the current state of knowledge on compensatory growth induced by Lys restriction in grow-finish pigs. The approach in the present review is threefold: 1) develop a database from peer-reviewed literature to standardize comparisons across the literature to characterize the occurrence of compensatory growth; 2) review the basis, types, factors, and dynamics involved in compensatory growth; and 3) provide practical considerations for compensatory growth under commercial conditions.

## DATABASE

### Literature Search and Selection Criteria

A literature search was conducted to compile published studies that directly or indirectly evaluated compensatory growth by having a period of Lys restriction followed by a recovery period of Lys sufficiency in the grow-finish phase. The search was performed via the Kansas State University Libraries under the CAB International database. The following terms were applied in the electronic-based search: (“lysine” OR “amino acid” OR “protein”) AND (“restriction” OR “limitation” OR “compensatory”) AND (“grow*” OR “finish*” OR “grow*-finish*”) AND (“pig” OR “swine”). Results were refined by language (“English”) and no restrictions were applied to the year of publication.

Publications were then individually evaluated for the following selection criteria: 1) peer reviewed; 2) conducted with pigs with an initial BW of at least 15 kg; 3) had a control group of “nonrestricted pigs” not subjected to a restriction period; 4) had a group of “restricted pigs” subjected to a restriction period induced by decreasing Lys alone, Lys and other amino acids, or crude protein (CP) in diets; 5) had a recovery period following the restriction period induced by providing the same diet to restricted and nonrestricted pigs; 6) presented growth performance data for restriction and recovery periods; 7) presented detailed diet composition; and 8) allowed ad libitum feed consumption. A total of 14 publications met all selection criteria and were included in the database.

### Database Development

Data collected from studies were entered in a spreadsheet template and included breed, sex, age, housing, number of pigs per pen, number of replicates, initial BW (kilograms), average daily gain (ADG; grams), average daily feed intake (ADFI; grams), and gain-to-feed ratio (G:F, grams per kilogram) for restriction, recovery, and overall periods, carcass leanness (percentage), carcass yield (percentage), longissimus muscle area traced between the 10th and 11th rib (square centimeters), and backfat thickness measured at the 10th rib (millimeters). For studies reporting feed efficiency as feed-to-gain ratio, the inverse proportion was calculated based on ADG and ADFI. For studies on fixed-time basis, the duration (days) of restriction and recovery periods were included. For studies on fixed-weight basis, the BW at the end of restriction and recovery periods were included. Then, data from all studies were converted to fixed-time basis to standardize comparisons among studies. To convert to fixed-time basis, the duration of restriction and recovery periods were derived by dividing the BW at the end of each period by the ADG of the respective period. The duration of restriction and recovery periods were converted to relative duration (%) by dividing the duration of each period by the overall duration in days and to a ratio of recovery to restriction duration by dividing the duration of recovery period by the duration of the restriction period in days.

Diets from all studies were reformulated by entering the diet composition into a spreadsheet-based formulator with NRC (2012) nutrient loading values for ingredients to achieve a common basis for dietary nutrient concentrations. The dietary nutrients obtained in as-fed basis included standardized ileal digestible (SID) Lys to calorie ratio (grams per megacalorie NE), CP (percentage), and neutral detergent fiber (NDF; percentage). The degree of Lys restriction (percentage) in the restriction period was estimated by dividing the dietary Lys to calorie ratio (grams per megacalorie NE) of restricted pigs by the dietary Lys to calorie ratio (grams per megacalorie NE) of nonrestricted pigs. Thus, the degree of Lys restriction (percentage) of restricted pigs is relative to the Lys level of nonrestricted pigs and based on the assumption that nonrestricted pigs were under no degree of Lys restriction in the restriction period.

Comparisons were conducted between restricted pigs and nonrestricted pigs within each of the 14 publications included in the database based on the number of treatments available for comparisons within each study. A total of 60 comparisons were conducted and three comparisons were excluded due to insufficient restriction as restricted pigs demonstrated similar or superior performance in the restriction period compared to nonrestricted pigs. Thus, the final database included 57 comparisons for all variables listed above, except for carcass leanness (9 comparisons), carcass yield (13 comparisons), longissimus muscle area (15 comparisons), and backfat thickness (20 comparisons), which were not available in all publications. For all variables listed above, the comparisons were performed as relative differences (%) between restricted pigs compared to nonrestricted pigs. The values of restricted pigs were divided by the values of nonrestricted pigs, multiplied by 100 to convert to relative values, and subtracted from 100 to indicate the relative difference from nonrestricted pigs:

$$\begin{array}{ll} & Relative\;difference\;\left(\%\right)\\ & \;=\left[\left(\frac{Values\;of\;restricted\;pigs}{Values\;of\;non-restricted\;pigs}\right)\times 100\right]\\ & \;-100 \end{array}$$

### Database Descriptive Summary

A summary of publications included in the database is presented in Table 1 and a descriptive summary of the database is presented in Table 2. The database descriptive summary is important to depict the characteristics of the data generated from the literature review and to understand the scope of inference of the present review.

**Table 1.** Summary of publications included in the database to evaluate compensatory growth following a period of Lys restriction in grow-finish pigs

Publication	Number of comparisons^1^	Breed	Sex	Number of pigs per pen	Number of pen replicates	Diet main ingredients	Average diet NDF, %	Average initial BW, kg	Average final BW, kg	Overall duration, d
Wahlstrom and Libal, 1983	15	Crossbred	Mixed	7	3–4	Corn soybean meal	8.7	26.9	101.1	98
Chiba et al., 1999	4	Crossbred	Mixed	1	4	Corn soybean meal	8.7	23.0	105.4	89
Smith et al., 1999	7	Crossbred	Gilt	2	5	Corn soybean meal	8.5	29.5	107.6	82
Fabian et al., 2002	3	Duroc	Mixed	2	4	Corn soybean meal	8.8	20.7	108.3	117
Chiba et al., 2002	4	Duroc	Mixed	2	8	Corn soybean meal	8.7	19.6	113.0	121
Fabian et al., 2004	1	Crossbred	Barrow	1	8	Corn soybean meal	8.8	21.2	107.8	102
O’Connell et al., 2006	4	Crossbred	Mixed	2	9	Barley wheat soy	12.7	34.9	95.9	68
Reynolds and O’Doherty, 2006	2	Crossbred	Mixed	11	9	Wheat barley peas soy	11.5	42.0	88.6	56
Skiba et al., 2006a	2	Crossbred	Gilt	1	6	Corn wheat barley soy	12.5	25.0	104.9	87
Yang et al., 2008	3	Crossbred	Mixed	4	4	Corn wheat soy	9.5	34.3	115.1	91
Main et al., 2008	3	Crossbred	Gilt	27	7	Corn soybean meal	8.1	32.8	116.4	103
Kamalakar et al., 2009	4	Yorkshire	Mixed	2	6	Corn soybean meal	8.8	22.7	111.0	91
Suárez-Belloch et al., 2015	3	Crossbred	Mixed	6	5	Corn wheat barley soy	11.0	26.3	124.8	115
Cloutier et al., 2016	2	Crossbred	Barrow	9	9–10	Corn wheat barley soy	10.7	26.6	103.4	85

^1^Comparisons were conducted between restricted pigs and nonrestricted pigs within each publication based on the number of treatments available for comparisons in each study. A total of 57 comparisons were conducted from 14 publications, except for carcass characteristics, which were not determined in all publications.

**Table 2.** Descriptive summary of the database used to evaluate compensatory growth following a period of Lys restriction in grow-finish pigs^1,2,3^

Item	Mean	Median	Minimum	Maximum	SD	*n* ^4^
Restriction period						
Initial BW, kg	27.7	26.7	18.2	52.0	6.5	57
Degree of Lys restriction, %^5^	33	33	7	59	14	57
Lys to calorie ratio, g/Mcal^6^	2.40	2.18	1.57	4.07	0.68	57
CP, %	14.9	13.9	11.0	21.5	2.8	57
Duration, d	39	37	28	75	11	57
Recovery period						
Initial BW, kg	56.7	49.9	32.2	78.4	12.4	57
Lys to calorie ratio, g/Mcal^6^	2.58	2.27	1.60	4.96	0.70	57
CP, %	15.6	14.7	11.7	23.4	2.7	55
Duration, d	55	59	26	86	18	57
Recovery to restriction ratio^7^	1.5	1.7	0.4	3.1	0.7	57
Restriction period growth performance						
ADG, % difference	−12.6	−11.5	−46.1	−1.3	8.5	57
ADFI, % difference	1.6	1.6	−17.8	26.4	7.1	57
G:F, % difference	−13.7	−14.9	−34.6	2.3	8.9	57
Final BW, % difference	−6.8	−5.6	−27.3	-0.6	4.8	57
Recovery period growth performance						
ADG, % difference	2.4	3.1	−14.9	16.4	6.4	57
ADFI, % difference	0.3	0.5	−17.0	22.5	6.7	57
G:F, % difference	3.6	2.8	−16.2	36.5	9.1	57
Final BW, % difference	−2.7	−2.5	−13.2	6.2	4.0	57
Overall period growth performance						
ADG, % difference	−3.4	−2.8	−19.9	15.3	5.9	57
ADFI, % difference	0.0	0.2	−13.8	14.2	5.1	57
G:F, % difference	−3.3	−2.4	−20.7	17.6	6.0	57
Carcass characteristics						
Yield, % difference	0.2	0.2	−3.3	4.4	1.9	13
Leanness, % difference	0.7	−0.4	−5.5	9.0	4.3	9
Longissimus muscle area, % difference	1.4	0.0	−12.2	22.2	9.5	15
Backfat thickness, % difference	0.1	0.6	−23.5	20.8	8.6	20

^1^Comparisons were conducted between restricted pigs and nonrestricted pigs within each publication based on the number of treatments available for comparisons in each study. A total of 57 comparisons were conducted from 14 publications, except for carcass characteristics which were not determined in all publications.

^2^For values listed as percentage difference, the comparisons were performed as relative differences between restricted pigs compared to nonrestricted pigs. The values of restricted pigs were divided by the values of nonrestricted pigs, multiplied by 100 to convert to relative values, and subtracted from 100 to indicate the relative difference from nonrestricted pigs.

^3^Restriction period is defined as a period of Lys restriction induced by decreasing Lys alone, Lys with other amino acids, or CP in diets offered to restricted pigs only. Recovery period is defined as a period of Lys sufficiency following the period of Lys restriction induced by providing the same diet to restricted and nonrestricted pigs.

^4^Number of comparisons conducted.

^5^Estimated by dividing the dietary Lys to calorie ratio of restricted pigs by the dietary Lys to calorie ratio of nonrestricted pigs.

^6^Expressed as a ratio of standardized ileal digestible Lys to net energy in grams per megacalorie.

^7^Estimated by dividing the duration of recovery period by the duration of restriction period in days.

On average, a degree of Lys restriction of 33% during a 39-d restriction period resulted in a decrease in ADG by 12.6%, G:F by 13.7%, and BW by 6.8% in restricted pigs compared to nonrestricted pigs. Following the restriction, a 55-d recovery period resulted in an increase in ADG by 2.4% and G:F by 3.6% in previously restricted pigs compared to nonrestricted pigs. However, on average, the improvement in growth performance in the recovery period was not sufficient to lead restricted pigs to a similar overall growth performance and final BW to nonrestricted pigs as there was approximately a 3% decrease in overall ADG, overall G:F, and final BW in restricted pigs compared to nonrestricted pigs. On average, carcass characteristics indicated a leaner carcass (0.7% greater carcass leanness and 1.4% greater longissimus muscle area) with virtually no difference in backfat thickness (0.1% greater backfat) or carcass yield (0.2% greater yield) in restricted pigs compared to nonrestricted pigs.

## BASIS OF COMPENSATORY GROWTH ACROSS SPECIES

**Figure 1.**
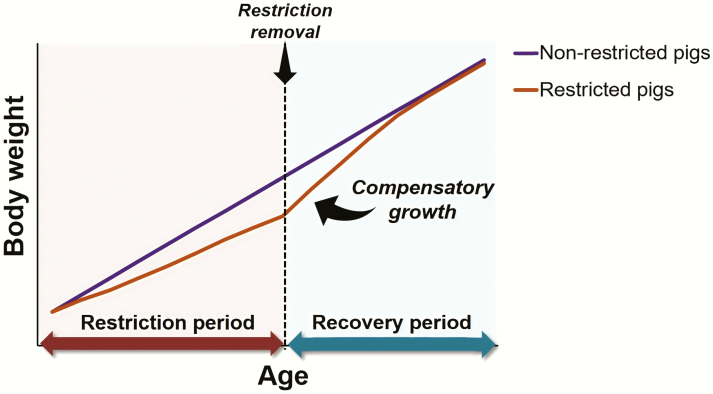
Representation of compensatory growth. The graph depicts a period of accelerated growth rate in restricted pigs compared to nonrestricted pigs following a period of growth restriction induced by nutritional deficiency.

Early studies by Osborne and Mendel (1916) described that animals with a decrease in weight gain due to nutritional restriction exhibit a subsequent rapid weight gain above normal growth rate under adequate nutrition (Fig. 1). The authors illustrate the physiological process as “curves of repair” alluding to the preservation of homeostasis as its central component (Osborne and Mendel, 1916; Wilson and Osbourn, 1960). During nutritional restriction, physiological maturation seems to proceed at a slower rate to preserve homeostasis (Ragsdale, 1934) but, then, under adequate nutrition, the growth rate of previously restricted animals seems to proceed at a faster rate proportional to the growth needed to reach maturity (Brody, 1926). The term “compensatory growth” proposed by Bohman (1955) is broadly used in the literature across species to refer to this growth phenomenon.

The pigs’ growth potential is determined by genotype and influenced by environmental and nutritional limitations (Gu et al., 1992; Schinckel and de Lange, 1996; Skinner et al., 2014). However, compensatory growth demonstrates that pigs have the capacity to achieve a rate of growth above the expected growth potential for a period of time. The pertaining question is: why not all pigs grow at the maximum rate throughout the growth period? Clues may be presented in other literature in other species. Particularly, in some species of animals in which adult size is important for fitness, reproduction, and survival, the acceleration of growth rate would allow animals to reach adult size at a younger age. However, there are often longevity costs associated with acceleration of growth in some species, including cellular damage, developmental errors, and senescence (Metcalfe and Monaghan, 2003). The intrinsic trade-off between benefits and costs of maximal growth rate varies within species, individuals, environment, and nutrition (Metcalfe and Monaghan, 2003). In the case of compensatory growth, the costs of acceleration of growth rate are often lower than the long-term consequences of previous nutritional restriction and impairment of adult size and weight (Metcalfe and Monaghan, 2001).

## TYPES OF COMPENSATORY GROWTH

Theoretically, pigs can exhibit complete or incomplete compensatory growth. Complete compensatory growth or “catch-up growth” refers to the occurrence of faster growth rate of previously restricted pigs compared to nonrestricted pigs that leads to the attainment of similar BW at a similar age (Skiba, 2005; Hector and Nakagawa, 2012). Incomplete compensatory growth refers to the occurrence of faster growth rate of previously restricted pigs compared to nonrestricted pigs, but the magnitude or duration of increase in growth rate is not sufficient to result in similar BW at a similar age (Skiba, 2005; Hector and Nakagawa, 2012).

**Figure 2.**
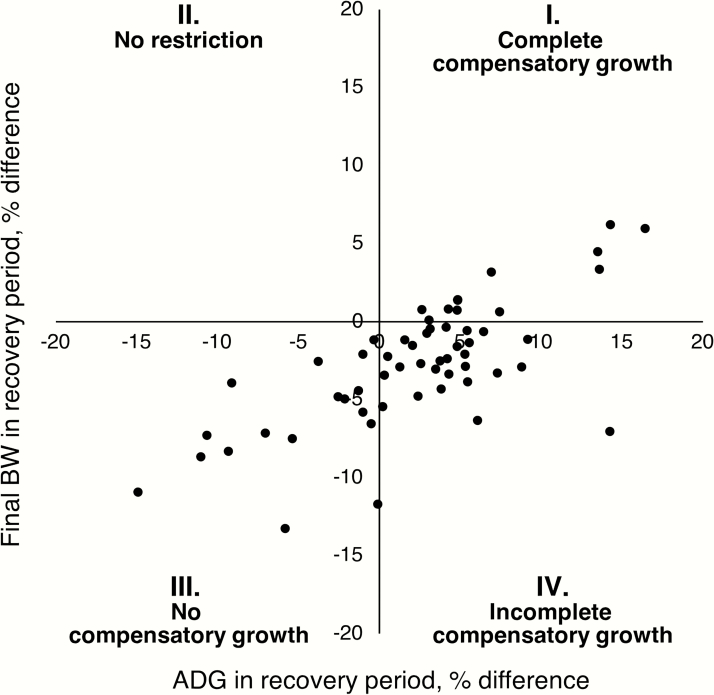
Plot of ADG in the recovery period against final BW in the recovery period as a relative difference between restricted pigs compared to nonrestricted pigs. The scatterplot depicts the distribution of all 57 database comparisons into four quadrants indicators of compensatory growth categories: I. complete compensatory growth due to an increase in ADG and final BW in restricted pigs compared to nonrestricted pigs; III. no compensatory growth due to a decrease in ADG and final BW in restricted pigs compared to nonrestricted pigs; and IV. incomplete compensatory growth due to increase in ADG but decrease in final BW in restricted pigs compared to nonrestricted pigs. In quadrant II, there is a decrease in ADG in the recovery period but an increase in final BW in restricted pigs compared to nonrestricted pigs, which means no restriction and, therefore, comparisons falling in quadrant II (3 out of 60) were excluded from the database.

The occurrence of complete and incomplete compensatory growth was assessed within the database. The ADG in the recovery period was plotted against the final BW in the recovery period as a relative difference between restricted pigs compared to nonrestricted pigs (Fig. 2). The scatterplot depicts the distribution of all 57 database comparisons into four quadrants. The comparisons falling in quadrant I indicate an increase in both ADG in the recovery period and final BW, which suggests that restricted pigs were able to exhibit complete compensatory growth and attain at least a similar BW to nonrestricted pigs at a similar age. Quadrant II indicates a decrease in ADG in the recovery period but an increase in final BW, which means that restricted pigs had an increase in ADG in the restriction period compared to nonrestricted pigs and, consequently, were not restricted. Because growth restriction is a primary requisite for compensatory growth to occur (O’Connell et al., 2006), comparisons falling in quadrant II (3 out of 60) were excluded from the database due to insufficient restriction. The comparisons falling in quadrant III indicate a decrease in both ADG in the recovery period and final BW, which suggests that restricted pigs were not able to exhibit compensatory growth. The comparisons falling in quadrant IV indicate an increase in ADG in the recovery period but a decrease in final BW, which suggests that restricted pigs were able to exhibit incomplete compensatory growth during the recovery period but not to attain a similar BW to nonrestricted pigs at a similar age.

The distinct patterns of complete, incomplete, or no compensatory growth within the database indicate that there are fundamental characteristics that place restricted pigs together in a category of compensatory growth and apart from others. This prompted the analysis of a number of factors by compensatory growth category.

## FACTORS AFFECTING COMPENSATORY GROWTH IN GROW-FINISH PIGS

The factors affecting compensatory growth have been clearly defined since the early literature about the subject (Wilson and Osbourn, 1960). There are generally four important factors: genotype, stage of growth at restriction, nature of nutritional restriction, and patterns of restriction and recovery. These factors alone or in combination are responsible for determining the occurrence and extent of compensatory growth. However, the complex interactions of these factors have not been well characterized and hinder the ability to accurately predict and control the occurrence and extent of compensatory growth in practice. An analysis of factors affecting compensatory growth within the database in the present review aims to aid in the clarification of some of these complex interactions.

### Genotype and Stage of Growth at Restriction

Genotype determines the potential for growth, protein deposition, and body composition in each stage of growth in swine (Gu et al., 1992). Compensatory growth can occur in contemporary lean or formerly fat strains of pigs (Hogberg and Zimmerman, 1978; de Greef et al., 1992; Chiba et al., 2002; Fabian et al., 2002), as well as in gilts, barrows, or entire males (Robinson, 1964; Smith et al., 1999; Fabian et al., 2004; Martínez-Ramírez et al., 2008). However, the compensatory growth response may vary based on the distinct genetic potential for growth, protein deposition, and body composition between strains and genders (Martínez-Ramírez and de Lange, 2007; Ruiz-Ascacibar et al., 2019). The genetic potential is relevant because the primary genetic aspects involved in compensatory growth in grow-finish pigs are the upper limit to protein deposition (Pdmax) and the body composition as a ratio of body lipid to body protein (Skiba, 2005; Martínez-Ramírez and de Lange, 2007).

The growth curve follows a nonlinear sigmoid shape in swine (Whittemore, 1986; Schinckel and de Lange, 1996). The BW increases with time until the inflection point of the sigmoid curve and plateau thereafter. The inflection point is determined by Pdmax. Until the inflection point, pigs are in an energy-dependent stage of growth as energy intake likely determines the rate of growth and protein deposition (Campbell and Taverner, 1988). After the inflection point, pigs are in a protein-dependent stage of growth as the inherent Pdmax signals the attainment of maturity and likely determines the rate of growth and protein deposition (Whittemore, 1986; Schinckel and de Lange, 1996). Early studies by Wilson and Osbourn (1960) emphasized that imposing an amino acid restriction at or after the inflection point during the protein-dependent stage of growth results in a lasting reduction in growth with no compensatory growth. In support, recent studies established that compensatory growth primarily occurs following amino acid restriction during the energy-dependent stage of growth and the extent of compensatory growth is dictated by Pdmax (Martínez-Ramírez et al., 2008, 2009). Thus, compensatory growth primarily occurs during the energy-dependent stage of growth before pigs reach Pdmax and, as a consequence, compensatory growth is more prone to occur in genotypes of relatively high Pdmax, which is characteristic of late-maturing, high lean growth potential pigs.

During the energy-dependent stage of growth, partitioning of energy intake is predominantly directed toward protein rather than lipid deposition. During the protein-dependent stage of growth, partitioning of energy intake is reversed and the ratio of protein deposition to lipid deposition decreases (Black et al., 1986; Quiniou et al., 1995). Studies by de Greef et al. (1992) suggested that partitioning of energy could be temporarily altered depending on the influence of nutritional restriction on body composition. In agreement, recent studies established that pigs have the ability to reach the target body composition represented as the ratio of body lipid to body protein following a period of amino acid restriction (Skiba et al., 2006b; Martínez-Ramírez et al., 2008, 2009). In that sense, pigs with increased body lipid to body protein ratio induced by amino acid restriction would have a preference for protein deposition over lipid deposition. This would occur for a certain period of time under adequate nutrition to reach the target body lipid to body protein ratio (de Greef et al., 1992; Martínez-Ramírez et al., 2008, 2009). Thus, compensatory growth seems to be driven by an inherent target body composition pigs aim to achieve.

### Nature of Nutritional Restriction

Compensatory growth can occur by imposing Lys restriction through diet formulation or through feed intake limitation. In the former, which is the scope of the present review, diets are formulated with low levels of Lys, Lys and other amino acids, or CP but are typically offered to pigs ad libitum. In the latter, diets are formulated with adequate levels of Lys but offered to pigs in limited amounts. Thus, there is a restriction in the intake of Lys, as well as other nutrients and energy. Depending on the nature of restriction, pigs have distinct changes in body composition, size of visceral organs, as well as voluntary feed intake and feed efficiency (Table 3). Thus, the nature of restriction determines important and distinctive aspects of compensatory growth response in grow-finish pigs (Skiba, 2005; Martínez-Ramírez and de Lange, 2007).

**Table 3.** Characteristic aspects of compensatory growth depending on nature of nutritional restriction^1,2^

Item	Lys restriction	Feed intake restriction
Restriction period		
Method of imposing restriction	Diet formulation	Intake limitation
Relative body protein composition	Lower	Higher
Relative body lipid composition	Higher	Lower
Visceral organs size	Similar	Lower
Recovery period		
Voluntary feed intake	Similar/ Higher	Higher
Gain efficiency	Higher	Similar/ Higher
Rate of body protein deposition	Higher	Similar
Rate of body lipid deposition	Similar	Higher
Visceral organs size	Similar	Higher

^1^Description of characteristics as lower, higher, better, or similar in regard to restricted pigs compared to nonrestricted pigs in restriction and recovery periods.

^2^Summarized from de Greef et al. (1992), Bikker et al. (1996a,b), Chiba et al. (2002), Fabian et al. (2002), O’Connell et al. (2006), Lovatto et al. (2006), Reynolds and O’Doherty (2006), Heyer and Lebret (2007), Kamalakar et al. (2009), Martínez-Ramírez et al. (2009), Chaosap et al. (2011), and Suárez-Belloch et al. (2015).

**Figure 3.**
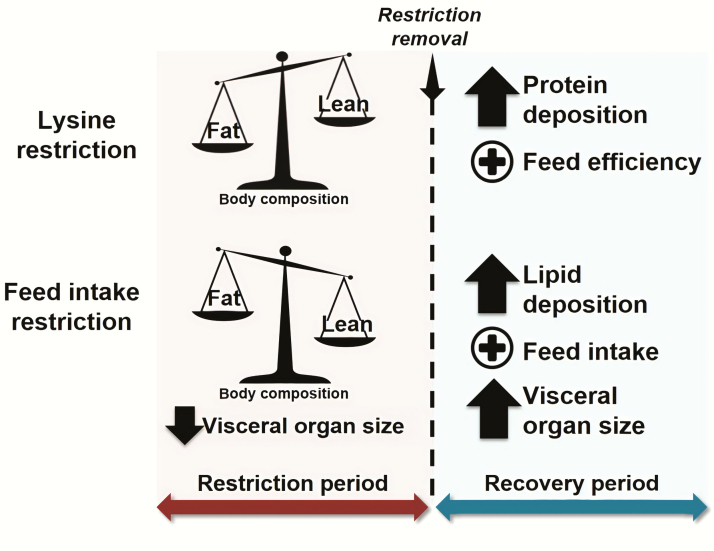
Representation of compensatory growth responses according to the nature of nutritional restriction: dietary Lys restriction or feed intake restriction. The figure depicts the differences in relative body composition during restriction and in composition of gain during compensatory growth according to the nature of nutritional restriction.

The primary difference in the compensatory growth response according to the nature of restriction lies in the composition of gain following restriction (Fig. 3; Skiba, 2005). In the case of Lys restriction, compensatory growth is driven by improvements in gain efficiency and primarily occurs by an increase in protein deposition in the carcass (de Greef et al., 1992; Chiba et al., 2002; Fabian et al., 2002; Martínez-Ramírez et al., 2008; 2009). In the case of feed intake restriction, compensatory growth is driven by an increase in voluntary feed intake and occurs by an increase in lipid deposition, as well as the size of visceral organs like liver, kidneys, and intestines and gut fill (Bikker et al., 1996a, 1996b; Lovatto et al., 2006; Heyer and Lebret, 2007; Chaosap et al., 2011). A similar restriction by limiting feed intake can be induced by diets with fibrous ingredients. In the case of high fiber diets, compensatory growth is driven by an increase in voluntary feed intake and lipid deposition, but the size of visceral organs and gut fill is already enlarged due to the fibrous content of the diet (Pond and Mersmann, 1990; Raj et al., 2005; Skiba et al., 2006a, 2006b).

The distinctive aspects of compensatory growth are related to the distinct body composition characteristics induced by nutrition in the restriction period (Fig. 3). Pigs under a period of Lys restriction typically have higher relative body lipid composition (de Greef et al., 1992; Kamalakar et al., 2009; Martínez-Ramírez et al., 2009; Suárez-Belloch et al., 2015), whereas pigs under a period of feed intake restriction have lower relative body lipid composition (Bikker et al., 1996a, 1996b; Lovatto et al., 2006; Heyer and Lebret, 2007; Chaosap et al., 2011) compared to nonrestricted pigs. Thus, in the recovery period and under adequate nutrition, protein and lipid deposition occur at different rates and ratios for pigs previously under a period of Lys restriction or feed intake restriction (de Greef et al., 1992). To reach a target body composition, pigs previously under Lys restriction direct resources to restore body protein reserves, whereas pigs previously under feed intake restriction direct resources to restore body lipid reserves (Skiba, 2005).

The target body composition also determines the main drivers of compensatory growth. In pigs previously under Lys restriction, gain efficiency is the primary driver and feed intake does not increase considerably due to appetite suppression mediated by body lipid stores and leptin (Chiba et al., 2002; Fabian et al., 2002; O’Connell et al., 2006; Reynolds and O’Doherty, 2006; Martínez-Ramírez et al., 2009). In pigs previously under feed intake restriction, feed intake is the primary driver to promptly increase energy intake (Bikker et al., 1996a, 1996b; Lovatto et al., 2006; Heyer and Lebret, 2007; Chaosap et al., 2011). The database analysis agrees on drivers of compensatory growth for pigs previously under Lys restriction, as pigs exhibiting complete compensatory growth have considerable improvements in gain efficiency but virtually no increase in feed intake (Table 4).

**Table 4.** Database analysis and characterization of compensatory growth categories in grow-finish pigs^1,2,3^

Item^4^	Complete compensatory growth	Incomplete compensatory growth	No compensatory growth
*n* ^4^	12	28	17
Restriction period			
Initial BW, kg	27.7	29.9	24.2
Degree of Lys restriction, %^5^	30	35	33
Lys to calorie ratio, g/Mcal^6^	2.53	2.38	2.34
CP, %	15.1	14.8	14.8
Duration, % overall duration^7^	37	45	44
Recovery period			
Initial BW, kg	53.6	58.6	55.7
Lys to calorie ratio, g/Mcal^6^	2.47	2.68	2.50
CP, %	15.2	16.0	15.3
Duration, % overall duration^7^	63	55	56
Recovery to restriction ratio^8^	1.8	1.4	1.5
Restriction period growth performance			
ADG, % difference	−6.3	−15.2	−12.8
ADFI, % difference	6.9	0.3	0.0
G:F, % difference	−11.4	−15.4	−12.6
Final BW, % difference	−3.2	−7.9	−7.4
Recovery period growth pserformance			
ADG, % difference	8.0	4.4	−5.1
ADFI, % difference	1.2	2.5	−3.9
G:F, % difference	9.7	2.4	1.2
Final BW, % difference	2.5	−2.6	−6.5
Overall period growth performance			
ADG, % difference	3.4	−2.9	−9.1
ADFI, % difference	1.2	1.8	−3.8
G:F, % difference	2.2	−4.3	−5.3
Carcass characteristics			
Yield, % difference	2.1	−0.2	−0.7
Leanness, % difference	−2.5	0.1	4.5
Longissimus muscle area, % difference	2.6	0.3	0.7
Backfat thickness, % difference	3.7	−0.1	−3.4

^1^Compensatory growth categories were defined by the distribution of all 57 database comparisons by plotting the ADG in the recovery period against the final BW in the recovery period as a relative difference between restricted pigs compared to nonrestricted pigs. Complete compensatory growth indicates an increase in both ADG and final BW in the recovery period. Incomplete compensatory growth indicates an increase in ADG in the recovery period but a decrease in final BW. No compensatory growth indicates a decrease in both ADG and final BW in the recovery period.

^2^For values listed as percentage difference, the comparisons were performed as relative differences between restricted pigs compared to nonrestricted pigs. The values of restricted pigs were divided by the values of nonrestricted pigs, multiplied by 100 to convert to relative values, and subtracted from 100 to indicate the relative difference from nonrestricted pigs.

^3^Restriction period is defined as a period of Lys restriction induced by decreasing Lys alone, Lys with other amino acids, or CP in diets offered to restricted pigs only. Recovery period is defined as a period of Lys sufficiency following the period of Lys restriction induced by providing the same diet to restricted and nonrestricted pigs.

^4^Number of comparisons conducted. Comparisons were conducted between restricted pigs and nonrestricted pigs within 14 publications for a total of 57 comparisons, except for carcass characteristics, which were not determined in all publications.

^5^Estimated by dividing the dietary Lys to calorie ratio of restricted pigs by the dietary Lys to calorie ratio of nonrestricted pigs.

^6^Expressed as a ratio of standardized ileal digestible Lys to net energy in grams per megacalorie.

^7^Estimated by dividing the duration of each period by the overall duration in days.

^8^Estimated by dividing the duration of recovery period by the duration of restriction period in days.

### Patterns of Restriction and Recovery

The patterns of restriction and recovery refer to both the nutrition and the duration of restriction and recovery periods. From the nutrition standpoint, both the degree of Lys restriction and the dietary Lys level are important. The degree of Lys restriction refers to the severity of Lys restriction in restricted pigs compared to nonrestricted, whereas the dietary Lys level refers to absolute Lys content. From the duration standpoint, both individual duration of restriction and recovery periods and the ratio of recovery to restriction periods are important. A recovery to restriction ratio below 1 indicates that the period of restriction is longer than the period of recovery, whereas a ratio above 1 indicates that the period of recovery is longer than the period of restriction.

The patterns of restriction and recovery determine the occurrence and extent of compensatory growth. The interactions among patterns are complex and have not been completely characterized but have already been well described (Wilson and Osbourn, 1960). While mild degrees of Lys restriction and/or short periods of restriction can cause minor effects in growth and not incite compensatory growth, severe degrees of Lys restriction and/or long periods of restriction can cause permanent stunting and prevent compensatory growth. Moreover, low Lys levels in recovery and/or short periods of recovery can prevent compensatory growth even following an ideal pattern of restriction, while high Lys levels in recovery and/or long periods of recovery cannot compensate for a severe pattern of restriction. Thus, the key to achieving compensatory growth seems to lie in finding ideal combinations and balances among all aspects involved in the patterns of restriction and recovery.

**Figure 4.**
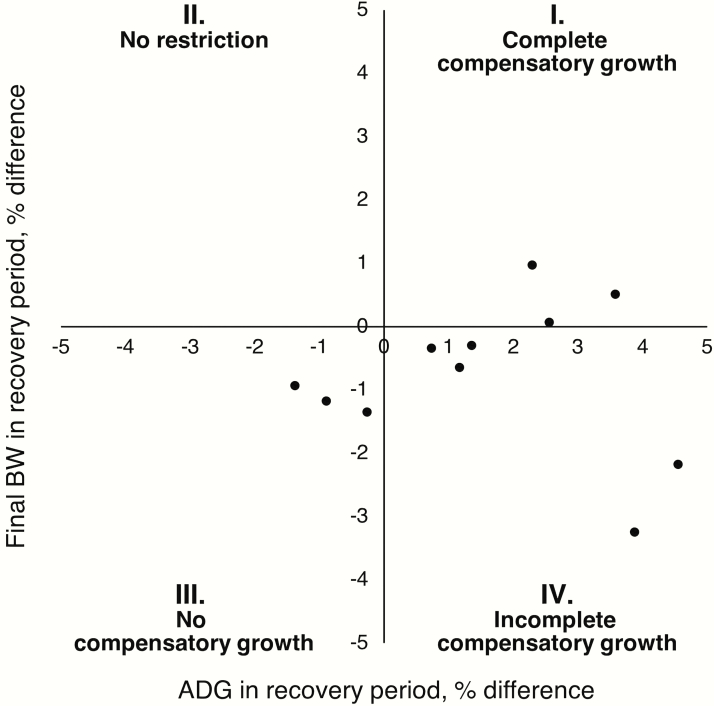
Database comparisons: differences in (a) BW as a relative difference between restricted pigs compared to nonrestricted pigs; (b) ADG as a relative difference between restricted pigs compared to nonrestricted pigs; and (c) Lys to calorie ratio as a ratio of standardized ileal digestible Lys to net energy, according to the category of compensatory growth in restriction and recovery periods.

The patterns of restriction and recovery according to compensatory growth categories defined in Fig. 2 are characterized in Table 4. The table summarizes differences and similarities between compensatory growth categories and aids in the identification of relevant aspects related to the occurrence of complete, incomplete, or no compensatory growth in grow-finish pigs. The differences in BW, ADG, and Lys levels in restriction and recovery periods by compensatory growth category are further illustrated in Fig. 4. Although the database analysis performed in the present review does not reflect cause-and-effect associations or is able to predict compensatory growth responses based on patterns of restriction and recovery, it provides important support for the characterization and conceptualization of compensatory growth in pigs.

First, the BW at restriction is similar across the compensatory growth categories as indicated by the initial BW at restriction and recovery periods. The BW at restriction is a relevant factor to observe beforehand because it determines the potential for compensatory growth to occur (Martínez-Ramírez et al., 2008, 2009). Compensatory growth primarily occurs during the energy-dependent stage of growth before pigs reach Pdmax. Pigs at lower BW at restriction are more prone to have compensatory growth because they are likely in the energy-dependent stage of growth, whereas pigs at heavier BW at restriction are less prone to have compensatory growth because they may be near their Pdmax and transitioning to the protein-dependent stage of growth (Möhn and de Lange, 1998).

The degree of Lys restriction across compensatory growth categories is substantial at approximately 30–35%. Pigs exhibiting complete compensatory growth were exposed to the least degree of Lys of restriction of 30% and fed diets with higher Lys level and CP content during restriction, whereas pigs exhibiting incomplete or no compensatory growth were exposed to more severe degrees of Lys restriction of 35% and 33%, respectively, and fed diets with lower Lys level and CP content during restriction. Also, pigs exhibiting complete compensatory growth were exposed to shorter restriction duration and longer recovery duration (37% and 63% of overall duration, respectively) than pigs exhibiting incomplete or no compensatory growth (44–45% and 55–56% of overall duration in restriction and recovery, respectively). However, in the recovery period, pigs exhibiting incomplete compensatory growth were fed diets with higher Lys level and CP content compared to pigs exhibiting complete or no compensatory growth.

Comparing the patterns of restriction and recovery, it is possible to identify important factors for complete, incomplete, or no compensatory growth in grow-finish pigs. The degree of Lys restriction and duration of restriction and recovery periods seem to be critical between complete and incomplete compensatory growth. If the restriction is too severe, too long, or both, and the recovery is too short, pigs seem to be more prone to exhibit incomplete over complete compensatory growth. The Lys level and CP content of diets in the recovery period seem to be critical between incomplete and no compensatory growth. If the Lys level and CP content of diets in the recovery period are too low, pigs seem to be unable to exhibit compensatory growth.

## DYNAMICS OF COMPENSATORY GROWTH IN GROW-FINISH PIGS

The physiological mechanisms involved in compensatory growth in pigs have not been completely elucidated. Characterizing the dynamics of compensatory growth allows understanding when compensatory growth occurs and what the potential underlying mechanisms of compensatory growth in pigs are.

### Body Composition and Carcass Characteristics

The rates of protein deposition and lean growth are increased in pigs following a period of Lys restriction (Chiba et al., 1999; Whang et al., 2003; Martínez-Ramírez et al., 2008). Recent models in rats suggest that both an increase in protein synthesis and a decrease in proteolysis contribute to greater protein deposition and lean growth but at distinct points in time (Ishida et al., 2011). The changes in the rate of body lipid to body protein ratio typically occur into the early recovery period (Reynolds and O’Doherty, 2006), with a decrease in proteolysis occurring only in the first days and an increase in protein synthesis prevailing throughout the entire period of compensatory growth (Ishida et al., 2011). Once protein stores have been replenished and target body composition is achieved, pigs return to normal protein and lipid deposition rates (O’Connell et al., 2006). Thus, the duration of compensatory protein deposition is determined by the amount of time required by the pig to achieve a target body composition (Martínez-Ramírez et al., 2008). There is consistent indication that compensatory growth induced by Lys restriction is not driven by changes in composition or the size of visceral organs (Fabian et al., 2002; Martínez-Ramírez et al., 2008, 2009; Kamalakar et al., 2009) or by increases in water deposition (Martínez-Ramírez et al., 2008).

The body composition of pigs during compensatory growth is often assessed by nitrogen balance (Fabian et al., 2004; O’Connell et al., 2006; Reynolds and O’Doherty, 2006; Ishida et al., 2012). Nitrogen utilization and nitrogen retention are improved while nitrogen excretion is decreased during compensatory growth (Fabian et al., 2004; Reynolds and O’Doherty, 2006; Ishida et al., 2012). The considerable improvements in nitrogen utilization and nitrogen retention have been described in restricted pigs from the restriction to recovery period (O’Connell et al., 2006), as well as compared to nonrestricted pigs (Fabian et al., 2004), which indicates an effort to replenish nitrogen reserves after restriction. Although the carryover effect of Lys restriction on nitrogen metabolism during compensatory growth is not well understood (Fabian et al., 2004; O’Connell et al., 2006), there seems to be a consistent improvement in the efficiency of Lys utilization for gain in pigs following a period of restriction compared to nonrestricted pigs (Whang et al., 2003; Fabian et al., 2004; O’Connell et al., 2006; Ishida et al., 2012; Cloutier et al., 2016). Because of the higher efficiency of Lys utilization in the recovery period, some authors suggest that the Lys requirements are also greater during compensatory growth (Whang et al., 2003), but this has not been confirmed experimentally.

The changes in body composition can be reflected in carcass characteristics. However, the influence of compensatory growth on carcass characteristics is variable and, in many instances, no effects are observed (Fabian et al., 2002, 2004; Reynolds and O’Doherty, 2006). The database analysis indicates distinct changes in carcass characteristics based on patterns of restriction and recovery and compensatory growth category (Table 4). Pigs exhibiting complete compensatory growth have less carcass leanness by 2.5% compared to nonrestricted pigs due to an increase in backfat thickness by 3.7% despite a 2.6% increase in longissimus muscle area, whereas pigs exhibiting incomplete compensatory growth have virtually no changes in carcass characteristics compared to nonrestricted pigs. The carcass composition data indicate that pigs exhibiting both complete or incomplete compensatory growth attempt to achieve a target body composition by adjusting fat and lean deposition as indicated by changes in longissimus muscle area alongside changes in backfat thickness or vice-versa.

### Metabolic Activity and Endocrine Status

Metabolic and hormonal indicators of metabolic activity and endocrine status in pigs are prone to be affected during compensatory growth (Skiba, 2005). Previous studies have focused on the description of metabolic changes during compensatory growth (Whang et al., 2003; Fabian et al., 2004; Yang et al., 2008), while more recent studies have focused on the endocrine regulation of compensatory growth (Martínez-Ramírez et al., 2009; Ishida et al., 2012).

A period of Lys restriction promotes a metabolic change in energy partitioning toward lipid deposition over protein deposition, with increases in triglycerides, cholesterol, and glucose concentrations and decreases in albumin and urea nitrogen concentrations in serum (Whang et al., 2003; Yang et al., 2008; Kamalakar et al., 2009; Suárez-Belloch et al., 2015). However, Lys restriction does not seem to have a long-term effect on metabolism as most serum metabolites rapidly return to normal concentrations during recovery (Fabian et al., 2004; Yang et al., 2008; Suárez-Belloch et al., 2015). The serum metabolite most often related to compensatory growth in pigs is urea nitrogen (Fabian et al., 2002; Whang et al., 2003; Yang et al., 2008). Urea nitrogen is often used as an indicator of amino acid catabolism and the efficiency of amino acid utilization (Coma et al., 1995). During compensatory growth, urea nitrogen is often low, which indicates an improvement in the efficiency of Lys utilization for growth (Fabian et al., 2002; Whang et al., 2003; Yang et al., 2008). However, there is no consensus about the use of urea nitrogen concentrations as an indicator of compensatory growth (Whang et al., 2003; Martínez-Ramírez et al., 2009).

The hormones involved in growth regulation and protein and lipid metabolism are the most likely to influence compensatory growth. A period of Lys restriction influences the endocrine system and promotes an increase in the concentration of growth hormone (GH) and leptin and a decrease in insulin-like growth factor I (IGF-I), IGF-binding proteins (IGFBP), cortisol, and corticosterone (Whang et al., 2003; Martínez-Ramírez et al., 2009; Ishida et al., 2012). Insulin-like growth factor I stimulates growth and protein synthesis (Sacheck et al., 2004), while cortisol and corticosterone stimulate proteolysis (Simmons et al., 1984). Leptin is a sensor of body adiposity and regulates lipid deposition (Barb et al., 1998). Thus, the endocrine status reflects the slow growth rate, low protein deposition, and high body lipid composition of pigs under Lys restriction. However, the concentrations of IGF-I, IGFBP, cortisol, and corticosterone immediately increase in recovery and in concert with improvements in growth rate and protein deposition (Martínez-Ramírez et al., 2009; Ishida et al., 2012). Moreover, GH and leptin remain at high concentrations in the immediate recovery to regulate protein and lipid deposition, respectively, and aid in the achievement of the target body composition (Martínez-Ramírez et al., 2009). Thus, there seem to be important endocrine components involved in compensatory growth in pigs.

The Lys level in the recovery period is also an important component of compensatory growth. However, it is often not possible to separate the influence of Lys level from endocrine components (Ishida et al., 2013). Recent in vitro models with myotubes have been conducted to determine the individual contribution of Lys level and endocrine components to compensatory growth (Ishida et al., 2013). Interestingly, the increase in the Lys level alone or the modulation of IGF-I and glucocorticoid levels alone were not able to influence the protein accumulation rate of myotubes. Thus, there seems to be a necessary combination of increased dietary Lys and modulation of endocrine status, indicated by IGF-I and glucocorticoid levels, to induce compensatory growth following a period of Lys restriction in pigs (Ishida et al., 2013). Further investigations in the area of metabolic and endocrine regulation of growth are warranted to characterize the influence and interaction of metabolites, hormones, and dietary components on compensatory growth in pigs.

## PRACTICAL CONSIDERATIONS RELATED TO COMPENSATORY GROWTH IN GROW-FINISH PIGS

The review of literature and database analysis provides robust evidence to support the occurrence of compensatory growth induced by Lys restriction in grow-finish pigs. However, as the database analysis in the present review mostly includes studies conducted under research conditions, the authors recognize there could be a concern about the occurrence of compensatory growth under field or commercial production conditions. Although the physiological aspects of compensatory growth are prone to occur under research or commercial conditions, there are additional factors under commercial conditions that could influence growth and, consequently, compensatory growth responses, for example, stocking density, number of pigs per feeder, environmental conditions, health challenges, and water quality and availability (Cornelison et al., 2018; Flohr et al., 2018; Wastell et al., 2018; De Oliveira et al., 2019).

**Figure 5.**
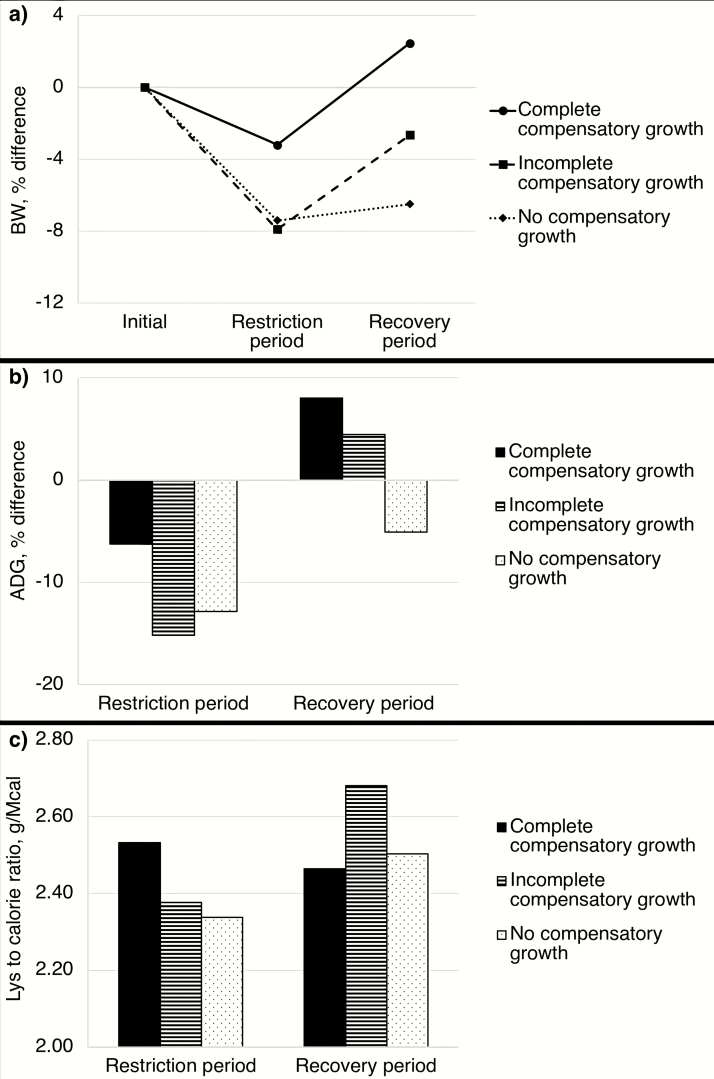
Field comparisons: plot of ADG in the recovery period against final BW in the recovery period as a relative difference between restricted pigs compared to nonrestricted pigs. The scatterplot depicts the distribution of 11 comparisons within four commercial studies into four quadrants indicators of compensatory growth categories: I. complete compensatory growth due to an increase in ADG and final BW in restricted pigs compared to nonrestricted pigs; III. no compensatory growth due to a decrease in ADG and final BW in restricted pigs compared to nonrestricted pigs; and IV. incomplete compensatory growth due to increase in ADG but decrease in final BW in restricted pigs compared to nonrestricted pigs. There were no comparisons falling in quadrant II, which means no restriction.

Recent studies with grow-finish pigs reared in commercial research conditions validate the database analysis and indicate that compensatory growth can occur in the field (Menegat et al., 2019). The same criteria and methods used to develop the database were applied to commercial studies. A total of 11 comparisons were conducted within four commercial studies between restricted pigs and nonrestricted pigs based on the number of treatments available for comparisons within each study, as previously described. To assess the occurrence of complete and incomplete compensatory growth within the commercial studies, the ADG in the recovery period was plotted against the final BW in the recovery period as a relative difference between restricted pigs compared to nonrestricted pigs (Fig. 5). The distribution of the field comparisons into quadrants depicts a similar pattern to the database comparisons, indicating the occurrence of complete, incomplete, and no compensatory growth. The growth patterns and the occurrence of compensatory growth throughout the grow-finish period are further illustrated in Menegat et al. (2019).

Thus, there seems to be an opportunity to exploit compensatory growth in grow-finish pigs raised in a commercial environment. In addition to recognizing the determining factors of compensatory growth, it is essential to consider the economic and practical implications of modifications in feeding programs or diet formulation to exploit compensatory growth. In economic scenarios of expensive dietary protein sources, relying on compensatory growth might be an economical approach. However, the economic feasibility of compensatory growth must be evaluated on a case-by-case basis, considering the costs of feeding programs and diet formulation, the potential improvements in feed usage and feed efficiency, and the projections in market weight under different market conditions. Moreover, overall nutrient use and efficiency to market must be evaluated to ensure that the Lys level in the recovery period is enough to allow compensatory growth but does not erase the savings in the Lys level in the restriction period. Finally, the practical feasibility of compensatory growth must be evaluated within the production system, considering the capability of providing accurate nutrient concentrations to all pigs. This might involve assessing the level of precision realistically achieved within the production system in terms of nutrient loading values of feed ingredients, feed manufacture, feed delivery, feed access, feed budget, average weight, and weight variation within a lot.

## CONCLUSIONS

Compensatory growth induced by Lys restriction is a measurable and repeatable response in grow-finish pigs as long as fundamental concepts are considered: 1) there are differences in types, rates, and extents of compensatory growth; 2) there are differences in physiological mechanisms of compensatory growth according to the nature of nutritional restriction, that is, Lys restriction through diet formulation or through feed intake limitation; 3) all factors that affect growth are also likely to affect compensatory growth, for example, health status, stocking density, and environmental conditions; and 4) genotype, stage of growth at restriction, nature of nutritional restriction, and patterns of restriction and recovery notably influence compensatory growth. The present review indicates that compensatory growth seems to be more likely if: 1) the degree of Lys restriction is around 10 to 30%; 2) Lys restriction is induced before pigs reach their maximum protein deposition (Pdmax); 3) the duration of Lys restriction is short (maximum 40–45% overall duration) and the duration of recovery period is long (minimum 55–60% overall duration); and 4) the Lys level in recovery is close to or above the estimated requirements. Compensatory growth can occur under commercial conditions and there seems to be an opportunity to exploit compensatory growth in grow-finish pigs to reduce feed cost and improve feed efficiency under certain market conditions.
